# Case Report: A case of superficial spreading malignant melanoma with sentinel lymph node metastasis misdiagnosed as “pigmented nevus”

**DOI:** 10.3389/fimmu.2026.1709633

**Published:** 2026-02-17

**Authors:** Xiaolin Bu, Jing Guo, Liwei Feng, Yeqiang Liu

**Affiliations:** 1 Department of Dermatology, Pudong Gongli Hospital, Shanghai University of Medicine & Health Sciences, Shanghai, China; 2 Postgraduate Training Base at Shanghai Gongli Hospital, Ningxia Medical University, Shanghai, China; 3 School of Gongli Hospital Medical Technology, University of Shanghai for Science and Technology, Shanghai, China; 4 Department of Pathology, Shanghai Skin Disease Hospital, School of Medicine, Tongji University, Shanghai, China

**Keywords:** atypical Spitz nevus, cutaneous malignant melanoma, immunohistochemistry, misdiagnosed, molecular genetic analysis

## Abstract

A 45-year-old woman presented with a black hemispherical plaque on her right lower leg that had developed three years ago without an obvious cause. The lesion gradually enlarged without pain or pruritus. After excision, histopathological examination revealed atypical Spitz nevus. At a dermatology specialty hospital, pathological review suggested superficial spreading melanoma. Immunohistochemistry revealed diffusely positivity for S100, Melan-A, Ki-67, P16, Sox-10 and HMB45. Molecular pathological testing revealed mutations in the TERT promoter and BRAF and CDKN2A/B genes. Extended tumor resection with an intraoperative sentinel lymph node biopsy identified SN2/2. Dissection of the right inguinal lymph nodes revealed melanoma metastasis in 10 of 14 lymph nodes, with Immunolohistochemistry showing diffusely positivity for Sox-10 and Melan-A. The final diagnosis was superficially spreading malignant melanoma with regional lymph node metastasis. Postoperative adjuvant therapy included systemic chemotherapy and targeted therapy. No recurrence was observed during the 2-year follow-up period.

## Introduction

Malignant melanoma (MM) is a malignant of melanocytes that occurs on the skin, eyes, ears, gastrointestinal tracts, leptomeningers and oral and genital mucous membranes. Its incidence has increased rapidly in the last decades globally, becoming one of the main causes of skin cancer-related death (1). Clinical practice demonstrated that early diagnosis and precise treatment were crucial for improving patient prognosis (2). However, clinical and pathological diagnosis of MM still faces numerous challenges, particularly in distinguishing it from benign or borderline pigmented lesions (such as Spitz nevus and their spectrum disorders).

Among the spectrum of differential diagnostic dilemmas, distinguishing atypical Spitz nevus from melanoma is especially difficult. Atypical Spitz nevus exhibits clinical and histological features intermediate between classic Spitz nevus and Spitz melanoma. Their biological behavior remains unclear, and lacks of consensus on diagnostic criteria, further complicating differential diagnosis. This diagnostic uncertainty poses a significant clinical risk: lesions with invasive potential may be misclassified as benign during initial evaluation, leading to delayed curative intervention and potentially adverse impacts on patient survival. Consequently, integrating clinical history and physical signs, histopathological morphology, immunohistochemical markers (e.g., p16, Ki-67, HMB-45), and molecular genetic features (e.g., mutations in BRAF, TERT promoter, CDKN2A/B genes) for precise differentiation has emerged as a core research focus for achieving accurate differentiation.

We reported a case initially diagnosed as “atypical Spitz nevus” that was subsequently reclassified as “superficial spreading malignant melanoma with regional lymph node metastasis” following comprehensive pathological review. By systematically analyzing the clinical progression, histopathological characteristics, immunohistochemical profiles, and molecular testing results, combined with the relevant review, we aimed to delineate the key diagnostic discriminants between atypical Spitz nevus and malignant melanoma. Furthermore, we explored the clinical utility of multi modal diagnostic approaches in resolving such challenging cases, and summarized the strategies for lymph node assessment and comprehensive management of misdiagnosed cases. Collectively, this study sought to provide evidence-based guidance for clinicians and pathologists, enhance the recognition of atypical melanoma variants, promote the standardization of diagnostic workflows and the implementation of individualized treatment strategies, and ultimately improve patient prognosis.

## Case presentation

A 45-year-old Asian woman presented to the dermatology clinic on April 10, 2023, with a three-year history of persistent plaque on her right lower leg. Three years earlier, the patient noticed a black hemispherical plaque on the right lower leg that gradually enlarged without symptoms. She denied any history of chronic diseases, family history of skin tumors, or trauma. The plaque underwent excisional biopsy, and histopathological diagnosis indicated an atypical Spitz nevus. Pathology consultation at the Shanghai Skin Disease Hospital suggested a high probability of malignant melanoma. The patient was subsequently transferred to our hospital and underwent extended tumor resection combined with sentinel and right inguinal lymph node dissection.

**Figure 1:**
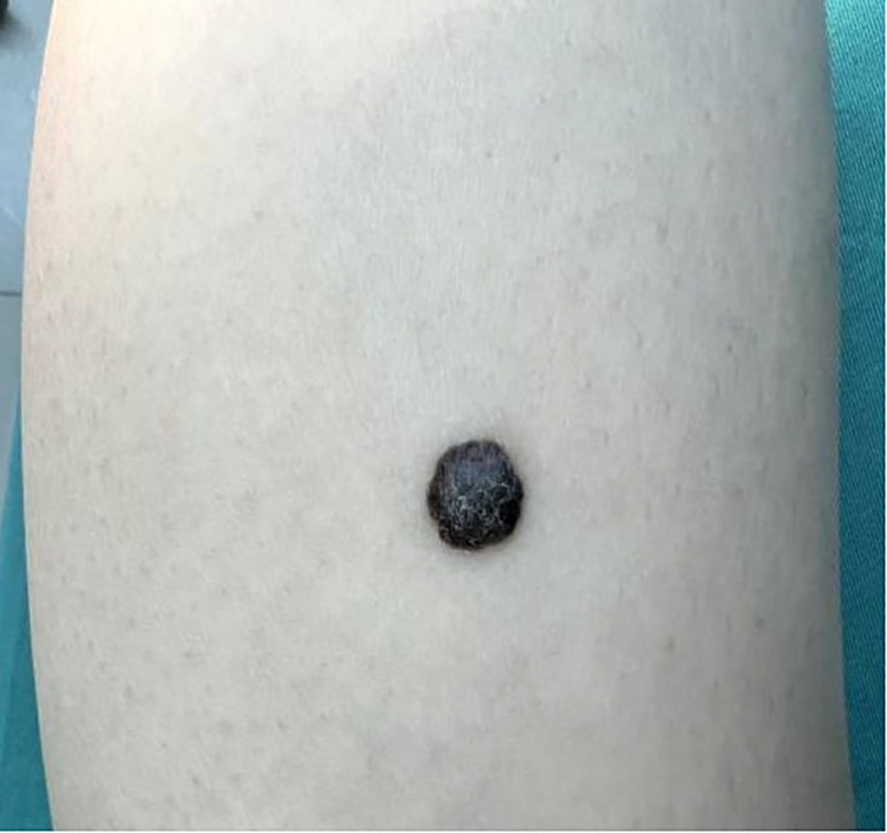
Skin lesion on the right lower leg of the melanoma patient. A black hemispheric plaque measuring 1.0×0.7×0.4 cm on the flexor aspect of the right lower leg. The lesion exhibited a rough, keratinized surface with scant scales, intact epidermis, firm texture, poor mobility, irregular yet well-demarcated margins.

Dermatological examination revealed a 1.0 × 0.7 × 0.4 cm black hemispherical plaque on the flexor calf of the right lower leg. The lesion exhibited a rough surface with keratinization accompanied by minimal scaling. The epidermis remained intact, with firm consistency, poor mobility and irregular but well-defined borders (**Figure 1**). Enlarged, moderately mobile, non-tender lymph nodes were palpated in the right inguinal region.

**Figure 2:**
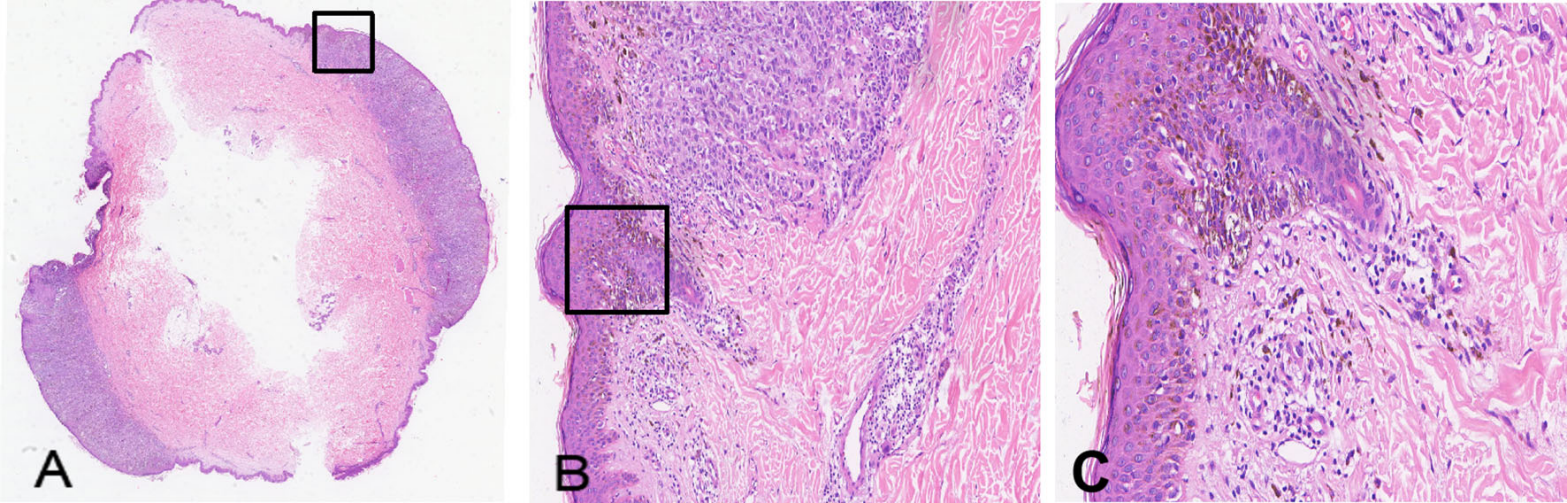
Histopathology of the skin lesion from the melanoma patient (H&E staining). **(A)** Cross-section of the hemispheric lesion (× 1); **(B)** Non-ulcerated lesion showing tumor cells spreading into the dermis (× 100); **(C)** Tumor cells arranged in sheets or nests, displaying abundant cytoplasm, variable nuclear sizes, and prominent nucleoli (× 200).

**Figure 3:**
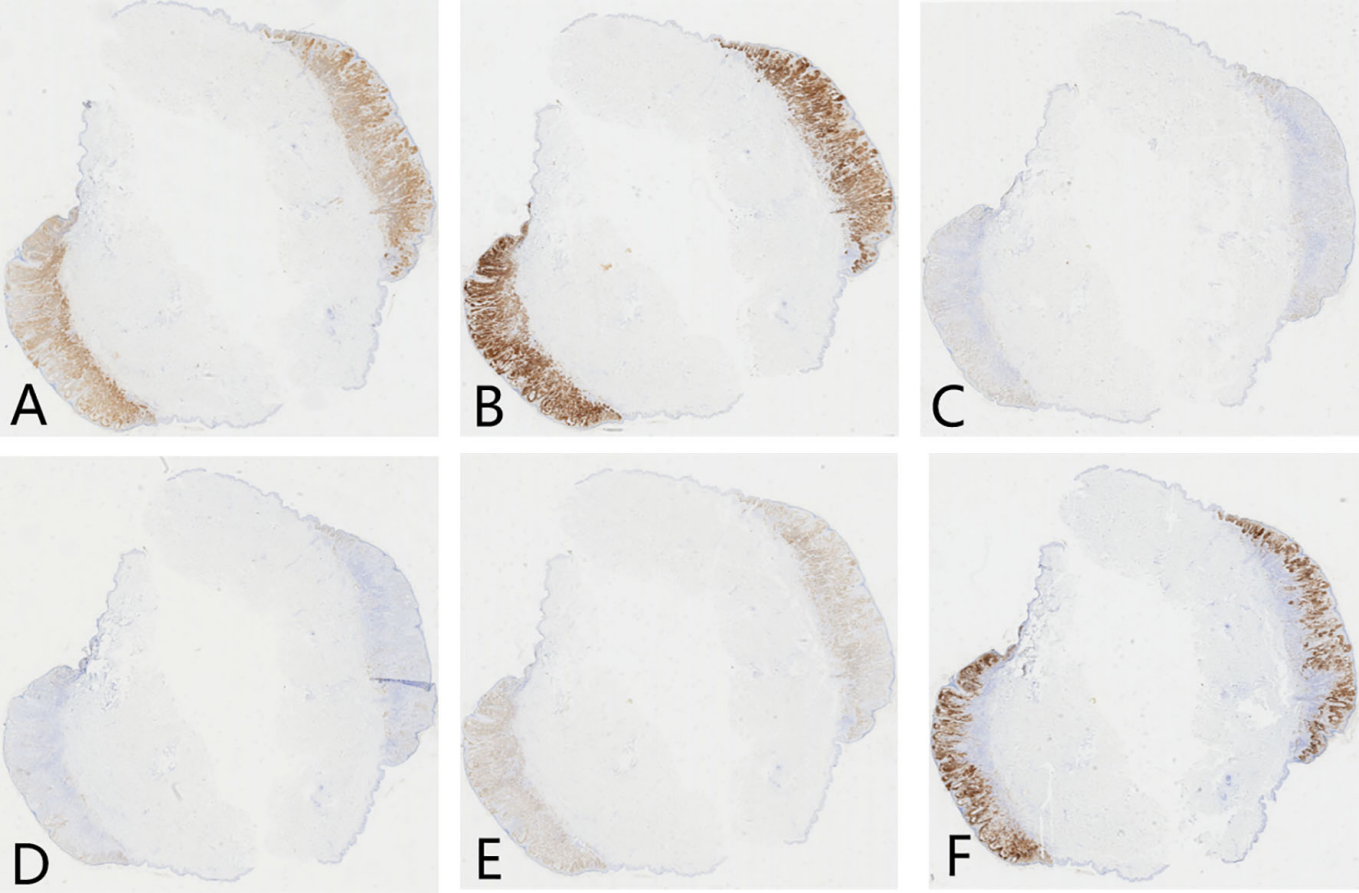
Immunohistochemistry of skin lesion in melanoma patient (× 100). **(A)** S100 protein positive (brown staining); **(B)** Melan-A positive (brown staining); **(C)** Ki-67 positive (brown staining); **(D)** P16 positive (brown staining); **(E)** Sox-10 positive (brown staining); **(F)** HMB45 positive (brown staining).

**Figure 4:**
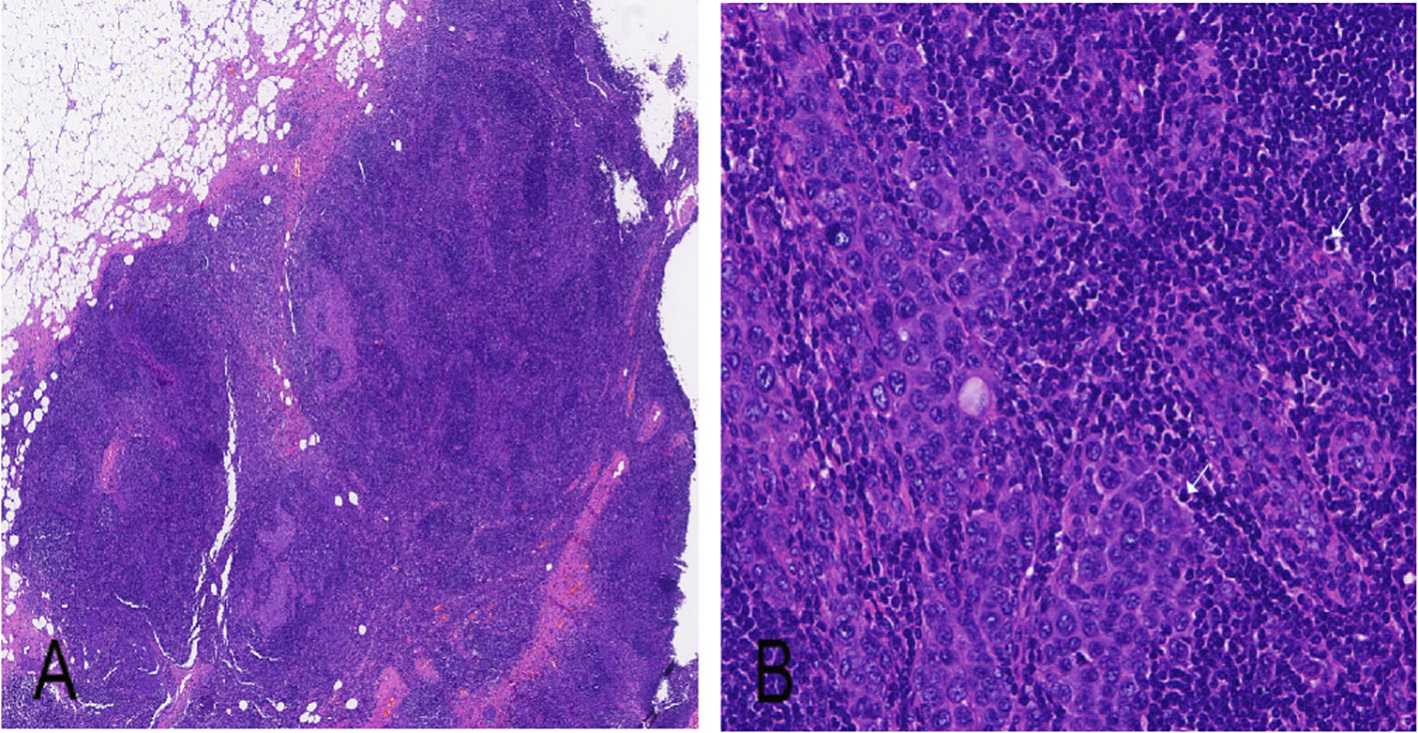
Histopathology of right inguinal lymph node (H&E staining). **(A)** Metastasis of melanoma cells within inguinal lymph node (× 40); **(B)** Melanoma cells exhibit large size, abundant cytoplasm, and large nuclei with prominent nucleoli (× 100).

**Figure 5:**
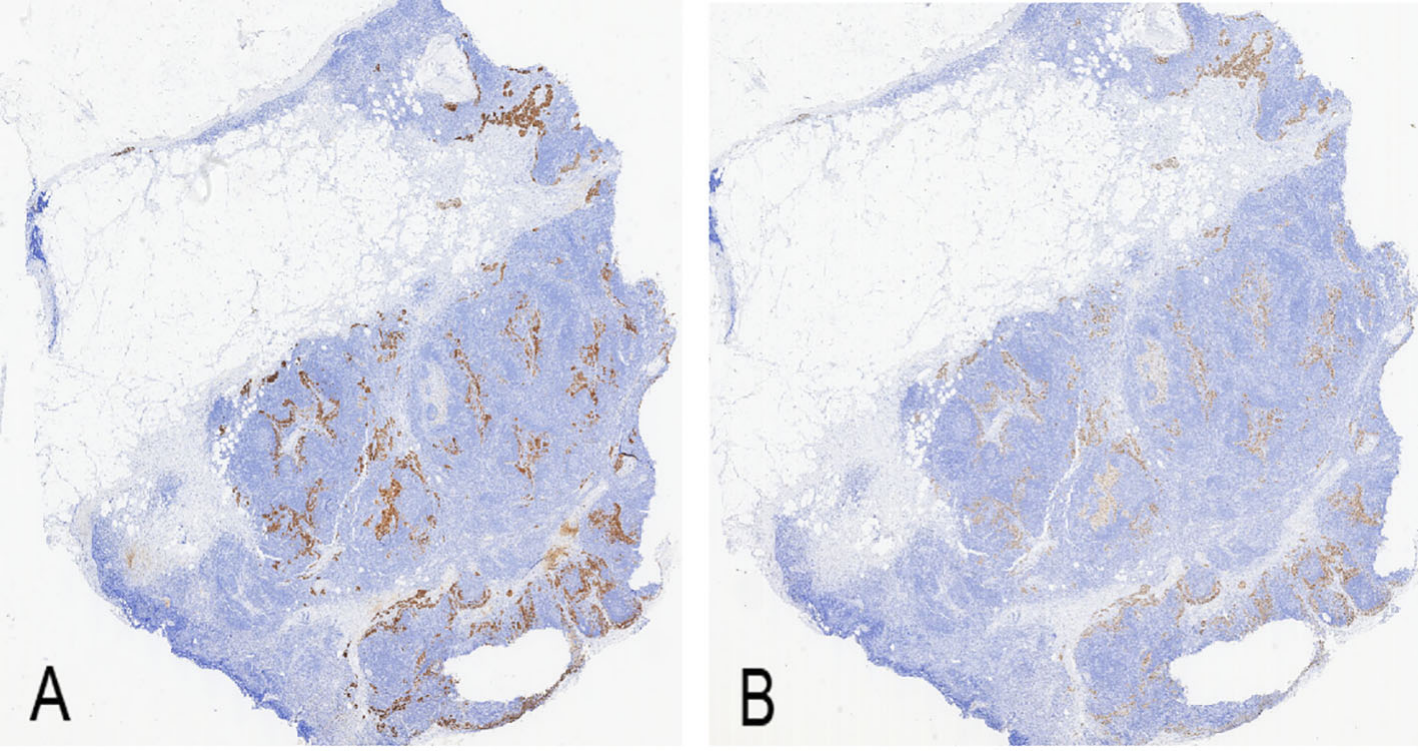
Immunohistochemistry of right inguinal lymph node (× 100). **(A)** Melan-A positive expression (brown staining); **(B)** Sox-10 positive expression (brown staining).

Ancillary testing: Histopathological examination of the skin lesion showed a symmetrical tumor architecture. Pleomorphic melanoma cells (round, oval, spindle-shaped, and polygonal) infiltrated the dermis in nests or sheets. These cells featured large hyperchromatic nuclei with prominent nucleoli, accompanied by intracytoplasmic deposition of melanin granules (**Figure 2**). The tumor invasion depth was Breslow 1.75 mm, Clark level IV. Immunohistochemistry revealed positivity for S100, Melan-A, Ki-67, P16, Sox-10, and HMB45 (**Figure 3**). Sentinel lymph node histopathology: SN (2/2). Of the 14 dissected inguinal lymph nodes, 10 demonstrated metastasis. Histopathology revealed irregular contours with disordered corticomedullary architecture and nested or sheet-like dense infiltration of metastatic melanoma cells (**Figure 4**). Immunohistochemistry demonstrated positivity for Sox-10 and Melan-A (**Figure 5**). Genetic analysis revealed mutations in the TERT promoter, *BRAF*, and *CDKN2A/B* genes. Imaging examinations: Abdominal color Doppler ultrasound showed that the liver, gallbladder, pancreas, spleen, and both kidneys exhibited normal morphology, size, and uniform parenchymal echogenicity, with no significant space-occupying lesions detected. A non-contrast chest CT scan showed no metastatic nodules in the lungs, abnormal mediastinal masses, or enlarged lymph nodes.

Diagnosis: Superficial spreading malignant melanoma with regional lymph node metastasis(pT_2a_N_3_M_0_, IIIC).

Treatment: Surgical tumor excision, regional lymph node dissection, and targeted therapy. Trametinib tablets (2 mg) were orally administered once daily. Dabrafenib mesylate (150 mg) was administered orally twice daily.

Outcomes: No evidence of recurrence or metastasis was observed during the 24-month follow-up period.

## Discussion

Cutaneous malignant melanoma is a highly malignant skin tumor originating from melanocyte (3). Atypical Spitz nevus is an intermediate lesion showing clinical and histopathological overlap with Spitz nevus and spitzoid melanoma, and its biological behavior remains unclear (4, 5). The evaluation of atypical Spitz tumors is challenging because of the partial overlap in clinical and histological features between entities (6); however, distinguishing benign from malignant lesions is crucial for treatment planning and prognosis assessment. Traditional diagnostic approaches primarily relied on morphological features or a limited number of immunohistochemical markers, reducing diagnostic accuracy and reliability possibly. In this case, the patient was initially diagnosed as atypical Spitz nevus; later, confirmed as MM after review. Its systematic diagnostic strategy offered significant advantages over most reported cases, providing valuable insights for reference.

In immunohistochemical (IHC) assessment, published case studies were predominantly limited to preliminary screening using 2~3 core markers (e.g., S100, Melan-A combined with Ki-67) (7, 8). Due to the limited scope of this approach, there was a risk of diagnostic bias. Additionally, the diversification of immunohistochemical markers may increase the risk of inconsistent results and make comprehensive interpretation more challenging. Therefore, selecting a reasonable combination markers is particularly crucial for accurate diagnosis and treatment. In this case, a comprehensive panel of immunohistochemical markers was employed, covering melanocyte differentiation (S100, Melan-A, Sox-10 and HMB45), cell proliferation (Ki-67) and cell cycle regulation (p16). Combined interpretation of p16, Ki-67 and HMB45 was central to the differential diagnosis. Previous studies indicated that diffusely p16 loss, a high Ki-67 proliferation index and diffusely, deep-level HMB45 positivity collectively provided highly specific evidence in support of a diagnosis of malignant melanoma (9). In this case, all of the aforementioned markers exhibited diffusely positive expression, which was unique among similar cases over the past five years. Most comparable cases exhibited negative or weakly positive results for some markers, with none demonstrating strong positive expression for all markers. This suggested a higher degree of malignancy in the tumor. The presence of sentinel lymph node metastasis in this case corroborated this finding further.

Over the past five years, we have reviewed 15 cases that were initially misdiagnosed as nevus, Spitz nevus or atypical Spitz nevus (**Table 1**). Genetic sequencing analysis was performed on 4 Spitz nevus, 2 atypical Spitz nevus, and 1 malignant melanoma. Gene fusions were detected in all cases (including LTK, NTRK1, ROS1, BRAF, and NTRK2). The malignant melanoma patient also exhibited BRAF gene mutation, which were absent in the atypical Spitz nevus and Spitz nevus. Previous reports on Spitz-like lesions primarily focused on single molecular mutation (e.g., BRAF mutations or TERT promoter mutations), with relatively weak diagnostic evidence (23, 24). In contrast, in this case, BRAF, TERT, and CDKN2A/B mutations were detected simultaneously. This composite molecular pattern was uncommon in published cases and provided clear diagnostic directionality and mechanistic explanatory value. Specifically, the BRAF mutation derived tumor proliferation by persistently activating the MAPK pathway. Common in classical adult melanoma but rare in benign Spitz nevus, it served as a key molecular marker for distinguishing tumor lineages. As the most frequent mutation type in MM, its occurrence shows a negative correlation with age, potentially explaining the relatively early onset in this patient (25). Furthermore, BRAF-mutant MM typically exhibited clear demarcation from surrounding unaffected skin tissue, consistent with the morphology of the lesion in this case. Most melanocytic tumors that mimic Spitz-like tumors morphologically but exhibit invasive behavior were essentially BRAF-mutant tumors that only mimic the phenotypic characteristics of Spitz-like tumors (26). TERT promoter mutations confer cellular immortalization by maintaining telomere length, often occurring after BRAF mutations (27). Their coexistence produces synergistic oncogenic effects. This mutation serves as an independent prognostic indicator for non-acral MM, closely linked to tumor aggressiveness and poor outcomes (28). Martins further suggested its prognostic significance was nearly equivalent to sentinel lymph node biopsy (29). CDKN2A/B mutation further synergistically promotes malignant progression by disrupting cell cycle regulation. Their high penetrance also provided valuable insights for familial risk assessment (30). The coexistence of these three mutations not only provided robust molecular evidence for malignant diagnosis but also rationally explained the highly invasive clinical behavior and occult widespread lymph node metastasis (10/14 nodes) observed in this tumor.

**Table 1:** Literature review of 15 cases misdiagnosed as “nevus/Spitz nevus/atypical Spitz nevus” in recent 5 years.

No.	Gender	Age	Lesion location	Initial diagnosis	Final diagnosis	Auxiliary examinations	Examination findings	Treatment	Follow up
1 (10)	Male	61	Left scapula	Atypical Spitz/ Reed nevus	Spindle cell/ Reed nevus of Spitz	NGS, FISH, IHC	NGS: SQSTM1::NTRK2 fusion; FISH: NTRK2 rearrangement; IHC: Pan-Trk diffusely strongly positive, HMB-45, MART-1, and tyrosinase positive, p16 focally weakly positive, BAP-1 expression retained, while PRAME, ALK-1, and ROS-1 all negative	Wide local excision	Not mentioned
2 (11)	Female	32	Right forearm	Atypical Spitz nevus	Spitz nevus	RNA sequencing, IHC	MLANA-BRAF fusion; IHC results not specified	Not mentioned	Not mentioned
3 (12)	Male	23	Left forearm	Melanocytic nevus	Spitz nevus	IHC	IHC: S100, Melan-A, SOX-10, BRAF all positive, HMB-45 negative	Surgical excision + Biopsy with a margin of 0.2 cm	Not mentioned
4 (13)	Male	15	Right thumbnail	Spitz nevus in atypical location	Subungual Spitz nevus	Histopathology, Dermoscopy	Histopathology: Junctional Spitz nevus; Dermoscopy: Serrated longitudinal black bands	Nail bed biopsy	Annual follow-up
5 (14)	Male	23	Right ankle	Nevus	Agminated Spitz nevus	RNA sequencing, FISH, IHC	RNA sequencing: GOPC(e8)-ROS1(e35) fusion; FISH: ROS1 rearrangement; IHC: ROS1 positive	Regular excision of individual lesions as they appeared (once per year)	Not mentioned
6 (12)	Female	22	Left lower limb	Compound Spitz nevus	Agminated Spitz nevus	RNA sequencing, FISH, IHC	RNA sequencing: GOPC(e4)-ROS1(e36) fusion; FISH: Unbalanced rearrangement; IHC: ROS1 partially negative	Regular excision of individual lesions as they appeared (6 excisions performed over the past 7 years)	Not mentioned
7 (15)	Male	6	Right buttock	Atypical Spitz nevus	Agminated Spitz nevus (with café-au-lait macule)	Histopathology, Dermoscopy	Histopathology: Spitz nevus; Dermoscopy: Irregular brown globules, pseudopods, regression areas	Complete removal achieved in two surgical sessions	Follow-up every 6 months, with no recurrence observed after 5 years postoperatively
8 (16)	Male	18	Left ear	Spitz nevus in atypical location	Compound Spitz nevus	Histopathology, Dermoscopy	Histopathology: Compound Spitz nevus; Dermoscopy: Polymorphous vessels, central white area, pseudoreticular depigmentation	Complete surgical excision	No recurrence or metastasis at 1-year follow-up
9 (17)	Male	40	Left scapula	Melanocytic nevus	Compound Spitz nevus	IHC	IHC: p16 diffusely positive, BRAF negative	Complete surgical excision	No postoperative recurrence or metastasis
10 (18)	Female	42	Right lower limb	Spitz nevus	Atypical Spitz tumor	RNA sequencing, IHC	RNA sequencing: LMNA::NTRK1 fusion; IHC: Pan-TRK diffusely cytoplasmic positivity, Melan-A, HMB-45 positive, p16 expression fully retained	Excision following diagnostic biopsy	Not mentioned
11 (16)	Female	17	Left foot	Spitz nevus	Atypical Spitz tumor	RNA sequencing, IHC	RNA sequencing: PRDX1::NTRK1 fusion; IHC: Pan-TRK diffuse cytoplasmic positivity, Melan-A, HMB-45 positive in both junctional and dermal components, p16 showing a checkerboard expression pattern	Excision following diagnostic biopsy	Not mentioned
12 (19)	Female	34	Left thigh	Spitzoid melanoma	Atypical Spitz nevus + ipsilateral nodal Spitz nevus	FISH, IHC	FISH: ROS1 protein diffusely strongly positive, ROS1 break-apart probe positive (rearrangement); Metastasis: Sentinel lymph node (inguinal) metastasis; IHC: Both skin and lymph node p16 diffusely positive, Ki67 shows dermal proliferative clusters but extremely low proliferation rate in lymph node, ALK and Pan-TRK weakly positive	Wide local excision + sentinel lymph node biopsy	Not mentioned
13 (20)	Male	34	Right cheek	Atypical dermal melanocytic proliferation (with Spitzoid features)	Malignant melanoma	IHC	IHC: SOX10 positive, HMB45 and PRAME showing patchy weak positivity, Ki-67 proliferation index 5–7%; Metastasis: No distant metastasis detected	Wide local excision	No recurrence or metastasin 9 months postoperatively
14 (21)	Male	17	Left thigh	Spitz nevus	Melanoma	IHC, Sentinel lymph node biopsy	IHC: MelanA positive; Ki-67 positive index 10%; Metastasis: Sentinel lymph node metastasis	Recurrence occurred 3 months after the initial surgical excision, and a re-excision with sentinel lymph node biopsy was performed	Recurrence occurred 3 months after the initial surgical excision
15 (22)	Male	25	Left upper limb	Nevus	Spitzoid melanoma	NGS, RNA-seq, IHC	NGS+RNA-seq: MYH9::LTK fusion, BRAF V600E mutation (nevus component only); IHC (epithelioid): Melan A, SOX10, HMB45 positive, p16 partially lost, PRAME weakly positive; IHC (nevus): BRAF V600E strongly positive, p16 positive, PRAME and HMB45 negative; Metastasis: Distant metastasis not mentioned	Plan for surgical excision with a margin of 0.5 cm	Not mentioned

The patient’s clinical presentation also provided crucial diagnostic clues. The skin lesion presented as a solitary, well-defined black nodule. Histopathological examination revealed a nest-like, symmetrically distributed proliferative lesion of the epidermis with sparse mitotic figures and mild cellular atypia in some cells. These features overlap significantly with those of atypical Spitz nevus, initially leading to a benign diagnosis. However, there are subtle differences between these two conditions. Spitz nevus and malignant melanoma exhibit differences in lesional characteristics: Spitz nevus predominantly occurs in children and adolescents, typically presenting as non-red, dome-shaped, elevated lesions <10 mm in diameter, often with scales or crusts on the surface (31), Whereas MM predominantly occurs in adults, typically presenting with skin lesions > 10 mm in diameter, and often exhibiting characteristic dark pigmentation (32). This case involved an adult patient with a black lesion measuring 10 mm in diameter, which aligned more closely with the clinical characteristics of melanoma. Although the ultrasound of the lymph nodes was normal in this case, lymph node dissection revealed positive metastasis. This was possibly because early stage melanoma metastases were often occult in the lymph nodes and typically lack morphological changes. Ultrasound demonstrated high specificity in preoperative assessment for guiding lymph node biopsy and dissection, but this was limited to patients with clinically apparent metastases. For occult metastases, ultrasound exhibited insufficient accuracy and sensitivity, with a high false-negative rate, potentially leading to missed diagnoses; thus, it cannot serve as a diagnostic criterion or definitive basis.

Early lymph node metastasis is a defining characteristic of malignant melanoma and a critical prognostic factor (33). Regional lymph node assessment is a critical factor in melanoma staging and provides important guidance for comprehensive treatment strategies (34). The sentinel lymph node (SLN) refers to the primary lymph node that drains lymphatic fluid from specific tissue areas of an organ, representing the first regional lymph node where primary tumors from that specific site metastasize. Sentinel Lymph Node Biopsy (SLNB) is a surgical procedure that determines tumor dissemination and metastasis by excising the SLN. This represents an optimal method for detecting occult lymph node metastasis. Therefore, it is crucial to determine the necessity of CLND for guiding melanoma staging and prognosis (35). SLNB is recommended for all patients with intermediate-thickness malignant melanoma. For SLNB-positive cases, CLND is recommended to achieve regional disease control (36). In this case, the tumor invasion depth was >1 mm, at 1.75 mm. This aligns with the 2018 clinical practice guidelines for SLNB issued by the American Society of Clinical Oncology and Society of Surgical Oncology (37). The lymphatic basin for biopsy was selected based on the distribution characteristics of the human skin lymphatic drainage corresponding to the primary tumor location. Lymphatic drainage in the extremities is relatively straightforward and fixed; the upper limbs drain into the ipsilateral axillary lymph nodes, whereas the lower limbs drain into the ipsilateral inguinal lymph nodes (38). The patient presented with a solitary skin lesion on the right lower leg and palpable enlarged, mobile, non-tender lymph nodes in the right inguinal region. Generally, unilateral lesions drain exclusively to ipsilateral regional lymph nodes (39). Therefore, lymph node dissection was performed in the right inguinal region, rather than in the popliteal fossa.

Pathologically, the diagnostic basis for malignant melanoma in this case includes the following: 1) Although the nevus cell nests demonstrate an overall symmetrical distribution, the dermo epidermal junction reveals characteristic shoulder phenomenon manifested by the unilateral peripheral extension of cell nests. This subtle architectural asymmetry within a symmetric context holds significant diagnostic value (40). 2) Despite irregular epidermal hyperplasia, focal areas show epidermal thinning, known as the “epidermal consumption phenomenon,” which suggests its significance for differential diagnosis (41). 3) Although the overall morphological features resembled those of a nevus, significant lymphocyte infiltration accompanied by an active proliferative state was observed at the base (42). This tumor-host interface reaction warrants serious consideration. Pathological examination of the lymph node biopsy specimen revealed metastatic melanoma cells exhibiting typical epithelioid morphology. These tumor cells were characterized by a large size, abundant cytoplasm, and enlarged nuclei with prominent nucleoli, resembling epithelial-derived tumor cells, which provided crucial evidence for a definitive diagnosis (43).

The patient was diagnosed as stage III malignant melanoma (pT2aN3M0) with lymph node metastasis. An integrated treatment strategy comprising “wide local excision, regional lymph node dissection, and postoperative adjuvant targeted therapy” was implemented to minimize recurrence risk. Because of BRAF V600E mutation, the postoperative adjuvant treatment selected the targeted regimen of Dabrafenib combined with Trametinib. This regimen, grounded in the COMBI-AD study, could significantly improve relapse-free survival in such patients, raising the 3-year relapse-free survival rate to 58%, which was a 34% increase compared to the placebo group (44). No recurrence was detected during the postoperative follow-up of 24 months of, further confirming the efficacy of this approach. Current adjuvant treatment options for stage III melanoma also include immune checkpoint inhibitors (e.g., PD-1 monoclonal antibodies) and high-dose interferon (45). Immunotherapy is particularly appropriate for BRAF wild-type patients or those intolerant to targeted therapy, offering the potential for durable responses. However, it is associated with a relatively slower onset of action and risks of immune-related adverse events, such as pneumonitis, hepatitis, and colitis (46, 47). Traditional high-dose interferon, due to its substantial toxicity and limited clinical benefit, has been largely superseded by newer agents (48). For patients with a high nodal tumor burden (N3), as in this case, aggressive postoperative systemic therapy is essential. In contrast, for those with a lower metastatic burden, active surveillance following complete resection may constitute a reasonable alternative. Clinical management should be individualized, taking into account the patient’s mutational profile, extent of disease, and overall clinical status.

In summary, malignant melanoma is well-known as the “great mimicker” due to its variable clinical and pathological presentations. Distinguishing it from benign pigmented lesions, such as Spitz-like nevi, remains a diagnostic challenge in clinical practice. Based on our experience of this case and a review of the relevant literature, we recommend maintaining a high level of vigilance for malignant melanoma in adult patients presenting with suspicious clinical features, such as lesions with a large diameter (especially >6 mm), changes in shape or color, or surface ulceration. This caution should persist even if the initial diagnosis leans towards benignity, in order to avoid missing malignant lesions due to initial diagnostic bias. When histological features exhibit atypical manifestations, such as structural asymmetry, epidermal depletion or active tumor-host interface reactions, promptly employ combined immunohistochemical testing (e.g. p16, Ki-67 and HMB-45 markers) and molecular genetic analysis (e.g. BRAF and TERT) for an in-depth evaluation to uncover potential evidence of malignancy. In clinical practice, establishing a multidisciplinary diagnostic workflow is essential. For cases with diagnostic uncertainty, prompt pathological consultation and sentinel lymph node biopsy should be performed to inform subsequent treatment strategy development.

## Data Availability

The original contributions presented in the study are included in the article/supplementary material. Further inquiries can be directed to the corresponding author.
